# Supplementary material for: Empirical methods for controlling false positives and estimating confidence in ChIP-Seq peaks
Source: BMC Bioinformatics. 2008 Dec 5;9:523. doi: 10.1186/1471-2105-9-523 (PMC2628906; doi:10.1186/1471-2105-9-523)
Supplement: Additional File 1 — A variety of html documents from the USeq web site detailing the available applications, their best usage, output file type descriptions, command line menus, etc. [file 1471-2105-9-523-S1.zip › USeqUserGuides/JavaDoc.zip!/JavaDoc/util/gen/SQL.html]

SQL


|  |  |  |
| --- | --- | --- |
| **Overview** \| **Package** \| **Class** \| **Use** \| **Tree** \| **Deprecated** \| **Index** \| **Help** | |  |
| **PREV CLASS**   **NEXT CLASS** | **FRAMES**    **NO FRAMES**     **All Classes** |  |
| SUMMARY: NESTED \| FIELD \| CONSTR \| METHOD | DETAIL: FIELD \| CONSTR \| METHOD |  |


---


## util.gen Class SQL

```
java.lang.Object
  util.gen.SQL
```

---

``` public class SQL extends java.lang.Object ```

Utility class to hold database and relevant fields and methods, some from Hall and Brown,
most from BioRoot. The following is a collection of stuff for interacting with a MySQL database.

**Author:**
:   nix

---

**Constructor Summary**

`SQL()`

`SQL(java.lang.String database,
java.lang.String user,
java.lang.String password,
java.lang.String dbURL,
java.lang.String dbDriver)`


| **Method Summary** | |
| --- | --- |
| `void` | `closeConnection()`             Closes the Connection and subsequently the Statement and ResultSet objects |
| `boolean` | `connect2Db(java.lang.String database, java.lang.String userName, java.lang.String password)`             Method to connect to the database and create a statement object |
| `java.sql.ResultSet` | `executeSQL(java.lang.String sqlQuery)` |
| `java.sql.ResultSet` | `executeSQLAdvance(java.lang.String sqlQuery)`             Execute and advance the ResultSet. |
| `boolean` | `executeSQLUpdate(java.lang.String sqlQuery)`             Fires SQL Update statement returning true if no problems were encountered. |
| `java.lang.String[]` | `fetchConcatMakeIdLast(java.lang.String sql, int numColumns, java.lang.String divider, int lengthCutOff)`             Returns a concatinate of the results where each String in the String[] represents a results table row where each column entry is separated by the divider String. |
| `java.lang.String[]` | `fetchMultipleCells(java.lang.String sql)`             Returns a String[] of the results. |
| `java.lang.String[]` | `fetchResultConcat(java.lang.String sql, int numColumns, java.lang.String divider, int lengthCutOff)`             Returns a concatinate of the results where each String in the String[] represents a results table row where each column entry is separated by the divider String. |
| `java.lang.String[]` | `fetchSingleColumn(java.lang.String tableName, java.lang.String columnName)`             Returns a String[] of the items in a particular column. |
| `java.lang.String` | `getCell(java.lang.String sql)`             Returns the first cell from an sql statement. |
| `java.sql.Connection` | `getConnection()` |
| `int` | `getLastInsertId(java.lang.String table)`             Returns the last inserted row auto increment number. |
| `java.sql.ResultSet` | `getResults()` |
| `int` | `getResultSetInt(java.lang.String columnName)`             Use to avoid a null return when attempting to fetch a ResultSet.getInt(). |
| `java.lang.String` | `getResultSetString(java.lang.String columnName)`             Use to avoid a null return when attempting to fetch a ResultSet.getString(). |
| `boolean` | `isNameUnique(java.lang.String tableName, java.lang.String name)`             Looks for a particular name in a table with a Name column. |

**Methods inherited from class java.lang.Object**

`equals, getClass, hashCode, notify, notifyAll, toString, wait, wait, wait`

**Constructor Detail**

### SQL

```
public SQL(java.lang.String database,
           java.lang.String user,
           java.lang.String password,
           java.lang.String dbURL,
           java.lang.String dbDriver)
```

---


### SQL

```
public SQL()
```


**Method Detail**

### fetchResultConcat

```
public java.lang.String[] fetchResultConcat(java.lang.String sql,
                                            int numColumns,
                                            java.lang.String divider,
                                            int lengthCutOff)
```

:   Returns a concatinate of the results where each String in the String[] represents a results
    table row where each column entry is separated by the divider String. If a particular column
    has no value, nothing is added, no double dividers. Use the lengthCutOff to limit the size of
    the Strings; a ... is added if it is trunkated. Returns new String[]{""} if no
    entries are present.

---


### fetchConcatMakeIdLast

```
public java.lang.String[] fetchConcatMakeIdLast(java.lang.String sql,
                                                int numColumns,
                                                java.lang.String divider,
                                                int lengthCutOff)
```

:   Returns a concatinate of the results where each String in the String[] represents a results
    table row where each column entry is separated by the divider String. If a particular column
    has no value, nothing is added, no double dividers. Use the lengthCutOff to limit the size of
    the Strings; a ... is added if it is trunkated. Returns new String[]{""} if no
    entries are present. The first query is assumed to be the id value and is always added to the end,
    even if the string was truncated. Thus the sql should look like "SELECT id, bla, bla FROM Table.."
    The result will look like bla,bla,id or bla, bla...,id
    This is a way of moving the ID to the end of the result yet trunkating in needed.

---


### fetchSingleColumn

```
public java.lang.String[] fetchSingleColumn(java.lang.String tableName,
                                            java.lang.String columnName)
```

:   Returns a String[] of the items in a particular column.
    Don't call on a table without the designated heading!
    Returns a new String[]{""} if no entries are present.

---


### fetchMultipleCells

```
public java.lang.String[] fetchMultipleCells(java.lang.String sql)
```

:   Returns a String[] of the results.
    Returns a new String[]{""} if no entries are present.

---


### closeConnection

```
public void closeConnection()
```

:   Closes the Connection and subsequently the Statement and ResultSet objects

---


### connect2Db

```
public boolean connect2Db(java.lang.String database,
                          java.lang.String userName,
                          java.lang.String password)
```

:   Method to connect to the database and create a statement object

---


### getResultSetString

```
public java.lang.String getResultSetString(java.lang.String columnName)
```

:   Use to avoid a null return when attempting to fetch a ResultSet.getString().

---


### getResultSetInt

```
public int getResultSetInt(java.lang.String columnName)
```

:   Use to avoid a null return when attempting to fetch a ResultSet.getInt(). Sorta stupid

---


### getLastInsertId

```
public int getLastInsertId(java.lang.String table)
```

:   Returns the last inserted row auto increment number.

---


### executeSQL

```
public java.sql.ResultSet executeSQL(java.lang.String sqlQuery)
```

---


### executeSQLAdvance

```
public java.sql.ResultSet executeSQLAdvance(java.lang.String sqlQuery)
```

:   Execute and advance the ResultSet. Good for retrieving a single table row.

---


### executeSQLUpdate

```
public boolean executeSQLUpdate(java.lang.String sqlQuery)
```

:   Fires SQL Update statement returning true if no problems were encountered.

---


### getConnection

```
public java.sql.Connection getConnection()
```

---


### isNameUnique

```
public boolean isNameUnique(java.lang.String tableName,
                            java.lang.String name)
```

:   Looks for a particular name in a table with a Name column. Throws
    error if no name column.

---


### getCell

```
public java.lang.String getCell(java.lang.String sql)
```

:   Returns the first cell from an sql statement.

---


### getResults

```
public java.sql.ResultSet getResults()
```


---


|  |  |  |
| --- | --- | --- |
| **Overview** \| **Package** \| **Class** \| **Use** \| **Tree** \| **Deprecated** \| **Index** \| **Help** | |  |
| **PREV CLASS**   **NEXT CLASS** | **FRAMES**    **NO FRAMES**     **All Classes** |  |
| SUMMARY: NESTED \| FIELD \| CONSTR \| METHOD | DETAIL: FIELD \| CONSTR \| METHOD |  |


---
